# Manifolds boosting severe acute malnutrition burden among children in and around Wolaita Zone, Southern Ethiopia: mini-review

**DOI:** 10.1186/s13104-018-3978-1

**Published:** 2018-12-06

**Authors:** Mulugeta Yohannis Kabalo

**Affiliations:** 1 College of Veterinary Medicine and Animal Science (CVMAS), University of Gondar (UoG), Gondar, Ethiopia; 2 School of Public Health, Wolaita Sodo University (WSU), P.O.Box: 126, Wolaita Sodo, Ethiopia; 3 DAB-Private Limited Corporation (PLC), Addis Ababa, Ethiopia

**Keywords:** Children, Household food insecurity, Severe acute malnutrition

## Abstract

**Objectives:**

Severe acute malnutrition (SAM) has been considered as the complex nutritional problem within developing countries. Alleviating its occurrence also exists in an anxiety. A series of studies were conducted to disclose evidences and documented by here author. Moreover key messages were abstracted with this review easing access of texts.

**Results:**

Due to pitiable sanitary practices 30% of cow milk had massive bacterial isolates like *Escherichia coli;* while usage of raw milk has been common do. Besides the mean severe household food insecurity was 6.5% and practice of family planning was 30%; whilst family size subsists as predictor for household food insecurity. The habits of exclusive breastfeeding, timely initiation of complementary feeding and apt complementary feeding were 78%, 34% and 11%, respectively with awareness as predictor. On the other hand SAM has been recognized as problem in children and treated mainly in outpatient therapeutic program by curative foods. Yet the provided foods were shared due to severe household food insecurity causing SAM recovery rate intolerable. So children get severely underfed by multidimensional determinants and need multifaceted strategies starting from awareness creation and alleviating household food insecurity.

## Introduction

“Studies” dictates citing a group of research papers published by here author. Overall eleven investigations were carried out in and around Wolaita Zone within Southern Ethiopia in the last 5 years. The key findings of the studies were appropriately documented in reliable scientific journals. Unfortunately the evidences in papers had links by indicating valid determinants of SAM burden among under five children. Furthermore the documented findings strengthen the concepts of “one health” and “multifaceted” nature of nutritional problems [1–11].

Among the subjects examined by the investigator; bacterial loads of dairy cow milk, household food insecurity and the state of family planning practices were a few to cite [2, 6, 9, 11]. Ahead of shown subjects; determinants of infant and young child feeding (IYCF) practices, occurrence of SAM in under five children and treatment procedures, treatment outcomes of SAM and its predictors were also areas investigated by the researcher [3–5, 7, 8, 10]. The investigations identified the burden of each problem and its key determinants in the studies setting. Besides, apt directions to alleviate the concerns were also indicated based on the study findings.

However as shown elsewhere findings had trends of key messages towards indicating versatile nature of severe nutritional problem in under five children. We recognize that nutritional problems are multifaceted, but devoid of adaptable intervention measures in developing countries including Ethiopia. So the impacts of interventions were hardly visible. Besides the findings from different research questions specify balanced messages querying multi-dimensional projects to alleviate nutritional problems among under five children.

Recognizing the merged documentation eases accessibility of texts and fastens the opportunities of appropriate interventions, the investigator decided to abstract key messages and point out costly intervention directions based on own findings. Therefore this short text was prepared from eleven research articles documented by the present author from 2013 to 2018. This document eases access of costly evidences for stakeholders and policy makers in favor of apt interventions of versatile nutritional problem in the setting.

## Main text

### Method

Overall 11 studies were carried out in and around Wolaita Zone, Southern Ethiopia by the investigator. Based on respective study questions; community based cross sectional, retrospective cross sectional and retrospective cohort study designs were implemented. Different sample sizes were calculated using the standard statistical formula and/or professional statistical packages still based on specific research questions. The sample sizes were ranging from 349 to 794 as per precise study queries and it was considered as representative for the source population.

Standard probability sampling technique was chosen for each study and/or design effect was used to compensate parallel effects. Data were collected by close supervision by apt field staffs. Then it was decisively managed in Excel, Epinfo, EpiData, SPSS and/or Stata statistical package software’s. Data were analyzed by univariate, bivariate and multivariate regressions as per need. The effect measures used were adjusted odds ratio and adjusted hazard ratio to fix the predictor variables after controlling likely confounders. Manuscripts were set and presented in varied workshops. Besides the well prepared manuscripts were disseminated in peer reviewed scientific journals. Still to ease access of evidences, this text was set by abstracting key findings of the records.

### Result and discussion

Overall 3% of under five children were severely malnourished based on the findings documented in the studies setting. Over 85% of those SAM children were treated in OTP which is part of community based management of acute malnutrition (CMAM). Once admitted, children receive ready-used therapeutic food weekly in view of their weight and other helpful therapies until discharge as per SAM management protocol [3].

#### Evidences from agricultural carves

Just about 30% of dairy cow milk had massive public health important bacterial isolates such as *Staphylococcus aureus*, *Streptococcus agalactiae* and *Escherichia coli.* Inadequate sanitation was the key predictor for bacterial loads in cow milk [11]. Besides household food insecurity was 38% and 4% of households were severely food insecure in the urban setting. Single household head, daily laborer household head, having dependent members in the household and poor monthly income had negative effect on urban household food insecurity [6]. Then again the burden of rural household food insecurity was identified as 38% and 9% of households were severely food insecure in the rural setting. Female headed household, larger family size, households head aged > 65 years, having lesser farm land and single household head had negative effect on rural household food insecurity [9].

#### Evidences from health service segments

The usage of long acting reversible contraceptives as family planning way was also identified as 30% and maternal education had effect on its usage [2]. Besides the practice of exclusive breastfeeding was 78%; affected by maternal education and awareness on the worth of breast milk for children [8]. As shown timely initiation of complementary feeding was also 34% and still maternal education, paternal education and postnatal care visit had ample effect on timely initiation of complimentary feeding [10]. In addition, apt complementary feeding practice was estimated as 11%; antenatal care visit and birth order were predictors of the practice [1].

On the other hand the level of SAM in children was well-known as public health problem in the studies setting and managed in CMAM mainly by outpatient form [3]. Yet the recovery rate was revealed as < 65% which is intolerable based on international sphere standard. Distance from OTP, type of malnutrition and provision of helpful therapies were key predictors for the outcomes. It was also documented as SAM children with oedema were more likely to recover and SAM admission was mostly in January to April in the studies area [3–5, 7].

#### The correlation of evidences boosting SAM in children

**Fig. 1:**
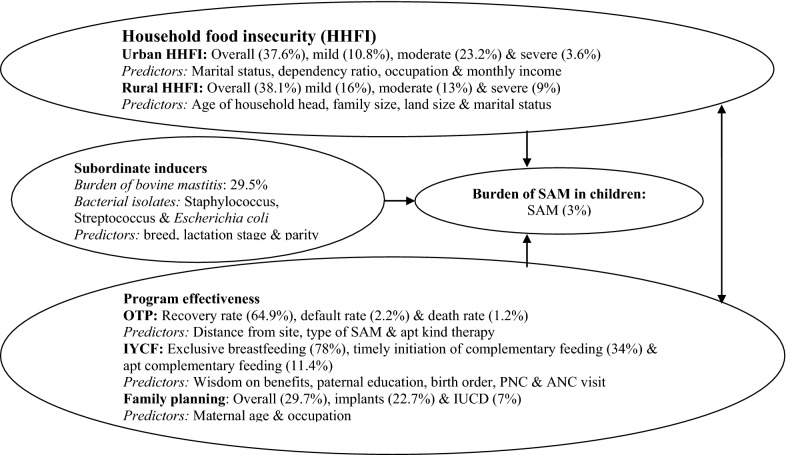
The partial view of complexity in nutritional problem in underfive children from own findings

The nutritious food option for children next to breast milk is cow milk, but using the foul raw cow milk has been common practice in the studies setting. So the pathogenic bacteria in cow milk were likely to cause diarrheal diseases which are instant cause of SAM in children. Besides household food insecurity has been known source of SAM incidence and also for its poor recovery rate due to sharing and selling of curative foods. Likewise apt IYCF practices were considered as vital for alleviating the incidence of SAM, but evidences show vast gaps in implementation of IYCF practices and the key obstacle for poor IYCF do was identified as still poor awareness. On the other hand CMAM is the easiest inclusive program for SAM management, but the validations shown as routine indicators were far out of international sphere standard yardsticks (Fig. 1).

The author bears in mind that other casuals also exist as nutritional problems are multifaceted; however children get severely malnourished perhaps owing to unhealthy consumption practices and/or due to severe household food insecurity and/or as result of poor IYCF practices based on the findings from here investigator. Besides SAM management by CMAM has been challenged by sharing and selling therapeutic foods perhaps because of household food insecurity and the habit of eating together. Yet family size was key predictor of household food insecurity, but the practice of family planning was silly boosting household food insecurity and the rate of SAM in children again (Fig. 1).

### Conclusions

The state of household food insecurity in urban setting argues calming food markets and creating job prospects. The sizeable severe household food insecurity in rural setting as well desires actions to pick up food production and productivity in the area. Besides awareness must be created on family planning do as family size enhances household food insecurity and be likely casual for SAM incidence among children. Moreover to reduce public health impacts, appropriate works needed in sanitary do above all on the usage of cow milk for children. The practice of exclusive breastfeeding was worthy but extra efforts darling in women education and awareness creation, as mothers think insufficiency of breast milk for their children. On the other hand timely initiation of complementary feeding and apt complementary feeding were also negligible. So nutrition specific care services and women education must be monitored. OTP admits large extent of SAM cases per year; however the routine performance indicators were intolerable. Thus focused outreach activities and monitoring of service provisions as per executive protocol be elective. Last but not least it’s wise that evidences by the present investigator update the multifaceted nature of nutritional problems inquiring versatile intervention measures in the field.

## Limitations of the present mini-review

Letting unnamed folds of SAM burden identified by other researchers secure and lack of follow-up studies incorporated by the investigator.

## Abbreviations

CMAM: community based management of acute malnutrition
IYCF: infant and young child feeding practice
OTP: outpatient therapeutic program
SAM: severe acute malnutrition

## References

[1] Areja A, Yohannes D, Mulugeta Y (2017). Determinants of appropriate complementary feeding practice among mothers having children 6–23 months of age in rural Damot sore district, Southern Ethiopia; a community based cross sectional study. BMC Nutr..

[2] Kabalo MY (2016). Utilization of reversible long acting family planning methods among married 15-49 years women in Areka town, Southern Ethiopia. Int J Sci Rep.

[3] Kabalo MY (2016). Management of severe acute malnutrition for children; principal consideration in outpatient therapeutic program in context of Ethiopia: review. Int J Health Rehabil Sci..

[4] Kabalo MY, Seifu CN (2017). Treatment outcomes of severe acute malnutrition in children treated within outpatient therapeutic program (OTP) at Wolaita Zone, Southern Ethiopia: retrospective cross-sectional study. J Health Popul Nutr.

[5] Kabalo MY, Shanka MM (2016). Seasonal variations of admission and survival status of children treated for severe acute malnutrition (SAM) at outpatient therapeutic program (OTP) in Wolaita Zone, Southern Ethiopia. Int J Collab Res Intern Med Public Health.

[6] Kabalo MY, Tantu AT, Gamebo TD, Sheno BK (2016). Household food insecurity and associated factors among households in Wolaita Sodo town, 2015. Agric Food Secur.

[7] Kabalo MY, Yohannes B (2018). Children with oedema recover better than those with severe wasting in outpatient therapeutic program at Boloso Sore district, Southwest Ethiopia. BMC Res Notes..

[8] Lenja A, Demissie T, Yohannes B, Yohannis M (2016). Determinants of exclusive breastfeeding practice to infants aged less than six months in Offa district, Southern Ethiopia: a cross-sectional study. Int Breastfeed J.

[9] Shone M, Demissie T, Yohannes B, Yohannis M (2016). Household food insecurity and associated factors in West Abaya district, Southern Ethiopia, 2015. Agric Food Secur.

[10] Yohannes B, Ejamo E, Thangavel T, Yohannis M (2018). Timely initiation of complementary feeding to children aged 6–23 months in rural Soro district of Southwest Ethiopia: a crosssectional study. BMC Pediatrics..

[11] Yohannis M, Molla W (2013). Prevalence, risk factors and major bacterial causes of bovine mastitis in and around Wolaita Sodo, Southern Ethiopia. Afr J Microbiol Res.

